# Safety of Leadless Pacemaker Insertion in Nonagenarians

**DOI:** 10.19102/icrm.2025.16053

**Published:** 2025-05-15

**Authors:** Jashan Gill, Ahmad Harb, Jobin Varghese, Rezwan Munshi, Michael T. Spooner

**Affiliations:** 1 Department of Cardiology, MercyOne North Iowa Medical Center, Mason City, IA, USA; 2 Department of Medicine, Northwestern Medicine McHenry Hospital, McHenry, IL, USA

**Keywords:** Leadless pacemaker, nonagenarian, pacemaker, safety

## Abstract

Increased age is associated with increased frailty and often worse postoperative outcomes. We sought to assess the safety of leadless pacemaker (LPM) insertion in the very elderly population. We queried the National Readmission Database for patients who underwent LPM insertion from 2017 to 2020. Patients aged ≥90 years were included in the nonagenarian group and compared to patients aged <90 years. Patient comorbidities were queried using the appropriate International Classification of Diseases, Tenth Revision, codes. We compared outcomes using multivariate logistic and linear regression, adjusting for patient comorbidities. At baseline, nonagenarians had higher prevalence rates of hypertension, a history of stroke, atrial fibrillation, atrial flutter, dementia, and hypothyroidism. The control group had more diabetes, coronary artery disease, chronic kidney disease, chronic pulmonary disease, oxygen use, coagulopathy, anemia, obesity, substance abuse, and chronic liver disease. Compared to controls, nonagenarians were found to have a shorter length of stay (2.5 days; *P* < .001); lower mortality (adjusted odds ratio [aOR], 0.7; *P* = .02); and lower rates of post-procedural cardiac arrest (aOR, 0.3; *P* = .03), mechanical ventilation (aOR, 0.4; *P* < .001), and vasopressor use (aOR, 0.6; *P* = .001). Nonagenarians were only found to have an increased risk of pericardial complications (tamponade, pericardiocentesis, hemopericardium) (aOR, 1.6; *P* = .02). There was no significant difference in 30-day readmissions (aOR, 0.97; *P* = .7), postoperative bleed (aOR, 0.84; *P* = .07), or stroke (aOR, 0.586; *P* = .1). Our study demonstrates that LPM insertion could be safe in the very elderly population. However, our study likely demonstrates survivorship bias, as patients in the nonagenarian group had fewer overall comorbidities. Despite adjustment for known comorbidities, there remain confounders that are difficult to account for. Age itself does not seem to be a risk factor for worse outcomes in this population.

## Introduction

Transvenous pacemakers (TVPs) are the gold standard for the management of bradyarrhythmias and cardiac conduction disorders.^1^ However, these lifesaving devices still possess limitations, with reported device-related complication rates of 8%–12%.^2,3^ These complications include pocket hematoma, lead dislodgement, venous obstruction, and device-related pocket or lead endocarditis.^4–7^ Leadless pacemaker (LPM) systems have emerged as an alternative to TVPs to mitigate these complications.^1^

The Micra™ transcatheter pacing system (TPS) (Medtronic, Minneapolis, MN, USA) and Aveir™ LPMs (Abbott, Chicago, IL, USA) are currently approved by the U.S. Food and Drug Administration.^8,9^ Post-approval studies have shown the safety of LPM systems, reporting 96% and 90% freedom from system- or device-related complications with Micra™ and Aveir™, respectively. Notably, the average age of participants in these studies was 70–75 years,^8,9^ with a small proportion of patients being >90 years of age. Several studies have shown that an age of >85 years is associated with a worse outcome in patients undergoing percutaneous transcatheter procedures.^10–12^ Furthermore, elderly patients have shown an increased risk for cardiac injury during LPM insertion.^9^ Given the lack of safety data in the nonagenarian population, we sought to specifically investigate the real-world safety of LPM insertion in this group of patients.

## Methods

### Study oversight

The protocol used aggregated de-identified data from a research network database and was deemed exempt from institutional review board (IRB) approval by the MercyOne North Iowa Medical Center IRB. The study’s findings comply with the Strengthening the Reporting of Observational Studies in Epidemiology (STROBE) guidelines for cohort studies.

### Data source

The study cohort was obtained from the National Inpatient Sample (NIS) covering the fourth quarter of 2017 through 2020. The NIS, part of the Healthcare Cost and Utilization Project (HCUP) and supported by the Agency for Healthcare Research and Quality, is one of the largest publicly accessible all-payer health care databases in the United States. It includes both weighted and unweighted hospital encounters each year. Researchers initially received unweighted data, constituting about 20% of national hospital inpatient data (databases). Using an algorithm provided by HCUP, the data were converted to weighted data, enabling the calculation of national estimates for analysis.

### Study population and outcomes

We conducted a retrospective cohort analysis of patients who underwent LPM insertion using appropriate International Classification of Diseases, Tenth Revision, codes. Patients aged ≥90 years were included in the nonagenarian group (study group) and were compared to patients aged <90 years (control group). The primary outcomes studied were in-hospital mortality, length of stay (LOS), 30-day readmission, and adjusted hospital charges. Secondary outcomes included post-procedural complications, including pneumothorax, hemothorax, acute kidney injury, cardiac arrest, pericardial complications, mechanical pacemaker complications, cardiac device infection, cardiogenic shock, bleeding, and stroke.

### Statistical analysis

The Pearson chi-squared test and Student’s *t* test were employed to evaluate categorical and continuous variables, respectively. To measure the risk of hospitalization outcomes, a two-step hierarchical multivariate regression model was used. This model included variables with *P* < .2 from a univariate regression analysis to make adjustments for baseline characteristics or comorbidities. The identified variables included in the multivariate regression analysis were hospital designation, hospital bed size, median income quartile based on ZIP code, and medical comorbidities **(Table 1)**. All statistical analyses were conducted using Stata Version 17 (StataCorp LLC, College Station, TX, USA).

**Table 1:** Patient Characteristics

	Age		
	<90 Years	≥90 Years	*P* Value
Total patients	22,937 (87.93%)	3147 (12.07%)	
Female patients	10,032 (43.74%)	1726 (54.85%)	<.001
Elective admission	19,457 (84.90%)	2830 (89.98%)	<.001
Charlson Comorbidity Index			
0	2327 (10.15%)	349 (11.09%)	<.001
1 (mild)	3543 (15.44%)	647 (20.54%)	
2 (moderate)	3751 (16.35%)	601 (19.10%)	
3 (severe)	13,316 (58.06%)	1551 (49.28%)	
Median household income national quartile for patient ZIP code			
Lowest 25%	5971 (26.33%)	623 (19.97%)	<.001
Lower-mid 25%	6065 (26.74%)	755 (24.20%)	
Higher-mid 25%	5732 (25.28%)	856 (27.42%)	
Top 25%	4909 (21.65%)	887 (28.41%)	
Hospital urban–rural designation			
Large metropolitan	13,013 (56.73%)	1763 (56.01%)	.941
Small metropolitan	9574 (41.74%)	1341 (42.59%)	
Micropolitan	311 (1.36%)	39 (1.25%)	
Rural	38 (0.17%)	5 (0.15%)	
Hospital bed size			
Small	1636 (7.13%)	309 (9.82%)	<.001
Medium	5028 (21.92%)	695 (22.10%)	
Large	16,273 (70.95%)	2143 (68.08%)	
History of stroke	3449 (15.04%)	537 (17.05%)	.034
Hypertension	18,275 (79.67%)	2666 (84.72%)	<.001
Diabetes mellitus	9615 (41.92%)	730 (23.19%)	<.001
Congestive heart failure (systolic or diastolic)	11,870 (51.75%)	1690 (53.69%)	.155
Peripheral artery disease	2042 (8.90%)	277 (8.79%)	.879
Coronary artery disease	10,759 (46.91%)	1376 (43.73%)	.009
Atrial fibrillation or atrial flutter	13,243 (57.74%)	1971 (62.62%)	<.001
Chronic kidney disease (stage 3 to end-stage renal disease)	10,013 (43.66%)	1260 (40.04%)	.007
Chronic obstructive pulmonary disease	5833 (25.43%)	580 (18.43%)	<.001
Tobacco use	8079 (35.22%)	779 (24.75%)	<.001
Anemia	7626 (33.25%)	923 (29.33%)	.002
Use of anticoagulation	8242 (35.93%)	1206 (38.31%)	.0615
Malnutrition	2171 (9.47%)	252 (8.02%)	.0637
Dementia	2724 (11.87%)	820 (26.05%)	<.001
Obesity	4822 (21.02%)	150 (4.76%)	<.001
Chronic liver disease	1863 (8.12%)	82 (2.62%)	<.001
Pulmonary hypertension	3620 (15.78%)	494 (15.71%)	.937

## Results

We identified 26,084 patients who underwent LPM insertion, with 3147 (12.07%) assigned to the nonagenarian group and 22,937 (87.93%) assigned to the <90 years of age control group. The baseline characteristics of both groups are summarized **(Table 1)**. A significantly greater proportion of patients in the nonagenarian group were female (54.58% vs. 43.74%; *P* < .001). Elective admissions were also more common in the nonagenarian group (89.98% vs. 84.90%; *P* < .001). The distribution across Charlson comorbidity categories showed that a smaller percentage of the nonagenarian patients fell into the highest comorbidity category (49.28% vs. 58.06%; *P* < .001). Socioeconomic factors, including median household income and hospital characteristics, were also examined. Nonagenarians were more likely to be in the top 25% income bracket (28.41% vs. 21.65%; *P* < .001) and less likely to be in the lowest 25% (19.97% vs. 26.33%; *P* < .001). There were no significant differences in hospital urban–rural designation or bed size between groups.

The nonagenarian group had greater prevalence rates of hypertension (84.72% vs. 79.67%; *P* < .001), history of stroke (17.05% vs. 15.04%; *P* = .034), dementia (26.05% vs. 11.87%; *P* < .001), and atrial fibrillation or atrial flutter (62.62% vs. 57.74%; *P* < .001). Conversely, they had lower rates of diabetes mellitus (23.19% vs. 41.92%; *P* < .001), coronary artery disease (43.73% vs. 46.91%; *P* = .009), chronic kidney disease (40.04% vs. 43.66%; *P* = .007), chronic obstructive pulmonary disease (18.43% vs. 25.43%; *P* < .001), tobacco use (24.75% vs. 35.22%; *P* < .001), anemia (29.33% vs. 33.25%; *P* < .001), obesity (4.76% vs. 21.02%; *P* < .001), and chronic liver disease (2.62% vs. 8.12%; *P* < .001). No significant differences between age groups were observed for other comorbidities, such as peripheral artery disease, congestive heart failure, use of anticoagulation, malnutrition, and pulmonary hypertension.

The results of the analysis comparing the primary outcomes between the nonagenarian and control groups are shown **(Table 2)**. Nonagenarians had a lower in-hospital mortality rate (3.0% vs. 5.1%; *P* = .019), LOS (6.7 vs. 10.9 days; *P* < .001), and hospital charges ($162,302 vs. $236,160; *P* < .001). The 30-day readmission rate also trended lower in the nonagenarian group but did not meet statistical significance (14.55% vs. 16.20%; *P* = .696). Regarding post-procedural complications, nonagenarians had a total of 168 (5%) post-procedural complications. There was an increased risk of pericardial complications (adjusted odds ratio [aOR], 1.596; 95% confidence interval [CI], 1.095–2.327; *P* = .0151) in the study group, which also demonstrated a decreased rate of cardiac arrest (aOR, 0.301; 95% CI, 0.104–0.870; *P* = .0267). There was no difference between the groups in rates of pneumothorax (aOR, 0.620; 95% CI, 0.192–2.008; *P* = .4256), hemothorax (aOR, 0.635; 95% CI, 0.183–2.209; *P* = .4754), acute kidney injury (aOR, 0.730; 95% CI, 0.165–3.235; *P* = .6783), pacemaker mechanical complications (aOR, 0.809; 95% CI, 0.500–1.307; *P* = .3858), cardiovascular implantable electronic device infection (aOR, 0.637; 95% CI, 0.342–1.187; *P* = .1556), or post-procedural stroke (aOR, 0.586; 95% CI, 0.303–1.133; *P* = .1121) **(Table 3)**.

**Table 2:** Outcomes

Outcome	>90 Years of Age	<90 Years of Age	Adjusted *P* Value
Length of stay	6.7 days	10.9 days	<.001
30-day readmission	458 (14.55%)	3715 (16.20%)	.696
Total hospital charges	$162,302	$236,160	<.001

**Table 3:** Post-procedural Outcomes

	Age n (%)				
Outcome	<90 Years 22,937 (87.93%)	≥90 Years 3147 (12.07%)	Adjusted Odds Ratio	95% Confidence Interval	Adjusted *P* Value
In-hospital mortality	1179 (5.1%)	94 (3.0%)	0.696	0.515–0.942	.018
Post-procedural pneumothorax	72 (0.31%)	5 (0.17%)	0.620	0.192–2.008	.426
Post-procedural hemothorax	105 (0.46%)	7 (0.22%)	0.635	0.183–2.209	.475
Post-procedural AKI	53 (0.23%)	3 (0.09%)	0.730	0.165–3.235	.678
Post-procedural cardiac arrest	140 (0.61%)	5 (0.15%)	0.301	0.104–0.870	.027
Pericardial complications	353 (1.54%)	65 (2.07%)	1.596	1.095–2.327	.015
Mechanical complication of pacemaker	391 (1.70%)	42 (1.32%)	0.809	0.500–1.307	.386
Infection of cardiac device	314 (1.37%)	19 (0.60%)	0.637	0.342–1.187	.156
Post-procedural cardiogenic shock	148 (0.64%)	8 (0.24%)	0.559	0.205–1.526	.257
Post-procedural stroke	220 (0.96%)	14 (0.46%)	0.586	0.303–1.133	.112
Total complications	1796 (7.8%)	168 (5.0 %)	0.689	0.599–0.792	<.001

*Abbreviation:* AKI, acute kidney injury.

## Discussion

Our study found that nonagenarians undergoing LPM systems had a statistically lower in-hospital mortality, LOS, and hospital charges compared to those aged <90 years, with a trend toward lower 30-day readmission rates. The overall complication rate for LPM implantation in nonagenarians was 5%, compared to a 7.8% rate in the non-nonagenarian group. Complications tended to be more likely to be pericardially related in nonagenarians (2%) and more likely to be cardiac arrest in the control group. There were no significant differences in other post-procedural complications between the age groups. While lower periprocedural mortality rates were seen in the nonagenarian group, we suspect this was due to more conservative patient selection in the higher-aged group. This was demonstrated by nonagenarians showing significantly fewer comorbidities compared to the non-nonagenarian group, as indicated by a lower percentage of patients in the highest Charlson Comorbidity Index category. Furthermore, nonagenarians had a higher percentage of elective admissions for LPM insertion compared to the non-nonagenarian group, supporting a lower procedural acuity and risk.

Previous studies have shown similar mortality and post-procedural complication rates to those of our study. Two previous studies evaluated mortality and complications after LPM implantation in non-octogenarian populations and showed similar results, with in-hospital mortality ranging from 5.0%–5.2%. The post-procedural complication rate was noted to be 8.6%. The authors in those studies noted a significant decrease in the overall mortality rate from 8.2% to 4.2% over a 2-year period from 2017 to 2019.^13^ Additionally, there was a concurrent decrease in overall complication rates, which fell from 20.6% to 13.0% during the same timeframe. The decrease in mortality rate over the study period was attributed to improved operator experience due to a learning curve with this new technology.^14,15^ Factors associated with in-hospital mortality included female sex, chronic kidney disease, heart failure, and malnutrition in one study.^16^ Furthermore, a more recent 2023 study from Hofer et al. evaluated LPM implantation in patients aged ≥85 years and found a high success rate of 98.6%, with a complication rate of 2.7%. The most common complication in that study was pericardial effusion, which was also noted to be more common in nonagenarians compared to non-nonagenarians, as in our study. Advanced age was identified as a risk factor for pericardial effusions in patients undergoing LPM implantation.^17^ Finally, a meta-analysis examining 18 studies with 2496 patients showed a complication rate of 3.1%, including pericardial tamponade, which occurred in 1.4% of patients. Other complications, such as pericardial effusion, device dislodgement, device revision, device malfunction, access site complications, and infection, occurred in <1% of patients.^18^

When comparing LPM to TVP implantation in elderly patients, similar mortality rates have been demonstrated in other studies. Pagan et al. evaluated the safety of LPM implantation in 183 patients aged ≥85 years by comparing them to 119 patients who had TVP implantation. LPM implantation was successful in all but three patients (98.4% success rate). These authors found no significant difference in procedure-related complications between the groups; the complication rate for LPM insertion was 3.3%. Specific complications included access site hematomas, pericardial effusion, and acute lead dislodgements.^15^ A separate study by Tachibana et al. included 27 patients, with a mean age of 90 years, who underwent LPM insertion and compared them with patients undergoing TVP insertion. The total complication rate was 7.4% in the LPM group. The noted complications were deep vein thrombosis and device dislodgement.^19^

Our study is the first to evaluate and find a decreased LOS and total hospital charges in nonagenarians compared to non-nonagenarians. We suspect this was due to more conservative patient selection in the nonagenarian group, given these patients had fewer comorbidities compared to the non-nonagenarian group. A previous study by Palmisano et al. comparing LPM to TVP implantation found that LPM implantation was associated with a shorter LOS. The mean LOS was 3.2 days for LPM, which was significantly lower than that in our study, which reported an average of 6.7 days. These data suggest that LPM may reduce the length of hospital stay compared to traditional pacemakers, potentially impacting overall hospital costs favorably.^20^ However, it is important to note that previous data have shown that the cost of hospitalization for LPM implantation was higher in 2019 and 2020 compared to 2016–2017, indicating a trend of increasing costs associated with LPM implantation over the years.^17^ It is important to note that the cost of the LPM ($19,990 manufacturer’s suggested retail price [MSRP]) is more than that of a single-chamber pacing system ($15,000 MSRP). However, the current Medicare reimbursement rate for the LPM (outpatient/inpatient, $15,940/$25,692) is significantly higher than that for a single-chamber TVP (outpatient/inpatient, $10,252/$12,990),^17^ which may be a consideration for clinicians.

Limitations of this study include the inherent deficiencies of national registry analyses; the NIS is an administrative database susceptible to documentation errors, coding errors, and misdiagnoses. Discharge-level coding relies on individual institutions; therefore, results may not be consistent across various centers. Due to the limitations with coding, we were unable to identify the indication for a pacemaker and which LPM system was used (Micra™ TPS vs. Aveir™). Furthermore, the database lacks context from each procedure, such as procedural techniques, number of device deployments, provider expertise, and procedure time. Finally, the dataset’s inherent limitations in capturing longitudinal outcomes and detailed clinical parameters constrain the depth of analysis regarding long-term prognosis and treatment outcomes.

## Conclusion

In this real-world representative sample, LPM implantation is safe in the very elderly population. The complication rate of LPM implantation in nonagenarians was 5%, which is similar to previous rates reported in both elderly and non-elderly cohorts. Although we found that nonagenarians had lower mortality compared to non-nonagenarians after adjusting for comorbidities and demographics, we suspect that the discrepancy was due to unknown confounders that cannot be accounted for. Notably, nonagenarians had fewer comorbidities and predominantly outpatient procedures. Chronological age alone should not exclude a patient from being considered for LPM. A more effective approach to clinical decision-making involves personalized medicine that incorporates the patient’s biological age, chronological age, and individual preferences.
